# TCP1 regulates PI3K/AKT/mTOR signaling pathway to promote proliferation of ovarian cancer cells

**DOI:** 10.1186/s13048-021-00832-x

**Published:** 2021-06-23

**Authors:** Huixi Weng, Xiushan Feng, Yu Lan, Zhiqun Zheng

**Affiliations:** Department of Ob & Gyn, Fujian Medical University Union Hospital, 29#, Xinquan Road, Gulou District, Fuzhou, 350001 Fujian China

**Keywords:** TCP1, Ovarian cancer, Prognostic biomarker, PI3K/AKT/mTOR signaling pathway

## Abstract

**Objective:**

TCP1 is one of the eight subunits of the TCP1 ring complex (TRiC) or the multi-protein mammalian cytosolic chaperone complex. TRiC participates in protein folding and regulates the expression of multiple signaling proteins and cytoskeletal components in cells. Although the clinical importance of its subunits has been clarified in various carcinomas, the function of TCP1 in ovarian cancer (OC) remains unclear. We aimed to identify the association between the expression of TCP1 and the development of epithelial OC (EOC) and patient prognosis, and explore the underlying mechanisms of TCP1 on the tumor progression of OC cells.

**Methods:**

TCP1 protein expression was tested in various ovarian tissues by immunohistochemistry, and the correlation between TCP1 expression and clinical physiologic or pathologic parameters of patients with EOC was analyzed. The relationship between TCP1 expression and the prognosis of patients with OC was investigated and analyzed using the Kaplan–Meier (KM) plotter online database. The expression level of TCP1 was then tested in different OC cell lines by Western blotting. Further, a model using OC cell line A2780 was constructed to study the functions of TCP1 in growth, migration, and invasion of human EOC cells. Finally, the possible regulating signaling pathways were discussed.

**Results:**

TCP1 protein expression in OC or borderline tissues was significantly higher than that in benign ovarian tumors and normal ovarian tissue. The upregulated expression of TCP1 in OC was positively associated with the differentiation grade and FIGO stage of tumors and predicted poor clinical outcomes. Compared with IOSE-80 cells, TCP1 protein was overexpressed in A2780 cells. TCP1 knockdown using shRNA lentivirus inhibited the viability of A2780 cells. Western blotting showed that the phosphatidylinositol-3 kinase (PI3K) signaling pathway was activated in the tumor invasion in EOC driven by TCP1.

**Conclusion:**

Upregulated TCP1 is correlated with the poor prognosis of patients with OC. The mechanism of cancer progression promoted by TCP1 upregulation may be linked to the activation of the PI3K signaling pathway, and TCP1 may serve as a novel target for the treatment of OC.

**Supplementary Information:**

The online version contains supplementary material available at 10.1186/s13048-021-00832-x.

## Introduction

EOC is the most common histotype of OC [1–3], which is a highly aggressive and lethal cancer among gynecologic cancers. Statistical data indicated that almost 70% of OC cases remain undetected until the advanced stage [4]. Currently, the standard treatment for advanced OC is primary cytoreductive surgery followed by combination chemotherapy using platinum and taxane [4–6]. Tumor recurrence may ultimately occur in approximately 75% of patients with advanced OC, and in 20% of the cases, the tumors become resistant [7]. Early diagnosis and new treatment strategies for OC have been around for the last three decades. However, effective anticancer drugs for the treatment of OC are still lacking, and extensive research in this regard is warranted [2, 8–10]. Therefore, it is necessary to clarify the underlying biological mechanism of EOC so as to develop effective novel anticancer drugs for it. A previous study reported that the expression of TRiC mRNAs increased over four-fold after OC cells became resistant to cisplatin after being exposed to it in an in vitro study [11].

TRiC is an important eukaryotic chaperonin [12–14]. It has double rings stacked back-to-back, with an empty central cavity [15]; each ring contains eight different, yet paralogous subunits (TCP1, CCT2, CCT3, CCT4, CCT5, CCT6, CCT7, and CCT8) [9, 16]. Each subunit has a molecular mass of approximately 60 kDa [12] and can recognize proteins of different polarities and hydrophobic subunits [17]. A recent study suggested that high mRNA expression of TCP1 was significantly associated with poor overall survival (OS) in patients with breast cancer [18]. TCP1 was essential for survival in breast cancer, and it was regulated by oncogene activation driven by PI3K signaling [19].

However, the functional role of TCP1 in EOC is still unclear. Therefore, we investigated the protein expression of TCP1 in EOC tissues and analyzed its prognostic value for patients with EOC to demonstrate the role of TCP1 in the growth and survival of OC cells. Subsequently, we knocked down TCP1 using related shRNA and evaluated its roles in the proliferation, invasion, and migration of EOC cells. Finally, we explored the possible mechanism underlying the function of TCP1 in the development of OC cells.

## Material and methods

### Tissue samples and patient data

The ethics approval for present study was obtained from the Ethics Committee of the Fujian Medical University Union Hospital. We collected 109 formalin-fixed paraffin-embedded ovarian tissue, including 13 normal ovarian tissue chips, 26 ovarian cystadenoma chips, 8 border ovarian tumour chips, and 62 ovarian malignant tumour chips from patients treated initially at the Fujian Medical University Union Hospital between 2016 and 2019. Relevant clinical parameter data were collected from the hospital medical record system and the definite histological diagnosis and grading came from the pathological reports. The clinical-stage were determined based on the International Federation of Gynecology and Obstetrics, 2009 (FIGO, 2009).

### IHC and quantitative analysis

Serial 3-μm sections from all samples were deparaffinized and rehydrated through xylenes and serial graded ethanol to water followed by antigen retrieval. These samples then were incubated overnight at 4 °C with TCP1 alpha primary antibody (Abcam Corporation; 1:200). The washed tissue samples were incubated with secondary antibody IgG (Merck Millipore; 1:300) for 30 min at room temperature (RT). Tissue slices were stained with 3,3’-diaminobenzidine and hematoxylin, and observed under an optical microscope. Finally, all images were analyzed integrated optical density (IOD) to calculate the average IOD /TCP1 positive staining area (μm ^2^) using Image-pro plus software.

### Survival analysis using KM plotter

The correlation between TCP1mRNA (Affymetrix ID: 222010_at) expression and survial rate of OC was analyzed using the Kaplan Meier plotter (http://kmplot.com/analysis). The cut-off date was set as overall survival (OS) and progression-free survival (PFS). The ruslt was presented with the hazard ratio (HR) and computed log rank *p*-value.

### Cell culture

The human EOC cell line A2780 and normal ovarian cell IOSE-80 were purchased from the Bena Culture Collection (Kunshan, Jiangsu Province, China) and cultured in 5% CO_2_ at 37 °C in dulbecco’s modified eagle medium (DMEM; Gibco) added with 10% fetal bovine serum (FBS; Gibco). Another EOC cell SKOV3 was obtained from Guangzhou Cellcook Biotech Company and cultured in 5% CO_2_ at 37 °C in McCoy’s 5a supplemented with 10% FBS. Cell line authentication by short tandem repeat (STR) profiling.

### Western blot assay

Total protein (20 μg/lane) was separated by polyacrylamide gel electrophoresis and then transferred to a PVDF membrane. Then, the membrane was blocked with 5% non-fat dry milk solution and incubated with the various primary antibodies at 4 °C overnight. Next day, the washed membrane using tris buffered saline tween (TBST) was incubated with horse radish peroxidase (HRP) conjugated secondary antibodies at RT for 2 h followed by visualized using chemiluminescent HRP substrate (Merck Millipore) on a Western blot imaging system. The band intensity was detected using Image Lab software. The protein expression was normalized to glyceraldehydes 3-phosphate dehydrogenase (GAPDH) expression. The primary antibodies used were anti-TCP1 alpha Rabbit Monoclonal (Abcam Corporation; 1:1,000), anti-mTOR Rabbit antibody (Abcam Corporation; 1:1,000), Akt antibody (CST Corporation, 1:1,000), phospho-Akt(Ser473) Rabbit mAb (CST Corporation, 1:1,000), and anti-GAPDH Mouse (TransGen Biotech, 1:2,000). Anti-mouse (Merck Millipore, 1:20,000) and anti-rabbit (Merck Millipore, 1:20,000) secondary antibodies were used.

### Construction of stable TCP1-knockdown cell line

The pLKO.1 Puro vector was used to construct lentiviruses for TCP1 RNA interference (shTCP1) and negative control (shCtr) experiments. The sequences targeting TCP1 were designed based on the human TCP1 gene (Table 1) and synthesized according to the pLKO.1 Puro vector specification. To prepare lentiviral particles, 8 μg of the shTCP1 vector (pLKO.1 Puro) and the packaging plasmids (5 μg pMDL, 3 μg pVSVG, and 2 μg pREV) were cotransfected into 293 T cells. The TCP1-NC group was transfected with negative lentivirus. Lentivirus-containing medium was collected after 48 h of transfection and used to culture A2780 cells. After 48 h of transfection, the medium was replaced with complete medium. Then, puromycin with a final concentration of 2.0 μg/mL was added for stable cell line screening for 72 h. Then, the survived cells were collected for TCP1 expression analysis. The stable cell lines were constructed in A2780/TCP1- negative control group (NC) and A2780/TCP1- knockdown group (KD), which were used for subsequent experimentation.

**Table 1:** The TCP1 shRNA sequences

Name	Sequence(5’-3’)
shTCP1 sense	CCGGGGTGTACAGGTGGTCATTATTCAAGAGATAATGACCACCTGTACACCTTTTT TG
shTCP1 anti-sense	AATTCAAAAAAGGTGTACAGGTGGTCATTATCTCTTGAATAATGACCACCTGTACA CC

### MTT assay

The proliferation of A2780/TCP1-NC and A2780/TCP1-KD cells was detected by MTT assay. The cells were firstly inoculated to 96-well plates (1,000 cells/well) and cultured in a humidified 5% CO_2_ incubator at 37 °C. Then, the plates were added with methyl thiazolyl tetrazolium (MTT, 0.5 mg/mL, 10 μL/well) at 24, 48, 72, and 96 h. After 4 h of normal culture, the supernatant was removed and purple formazan crystals were dissolved using a 150 μL dimethyl sulfoxide (DMSO) solution. The plate oscillated for 10 min at RT. The optical density at 490 nm (OD490) of each well was measured by microplate reader using wells without cells as blanks. The cell viability curve was drawn by the abscissa of the time point and the ordinate of OD value. Each experiment was performed in triplicate.

### Colony formation assay

Infected cells were routinely harvested, resuspended, and then placed in 6-well plates (1000 cells/well) to analyze cell colony formation. After 10 days of incubation with each 3-day medium changes, the surviving cells were washed using cold phosphate buffered solution (PBS), fixed by 4% polyformaldehyde, and dyed with 1% crystal violet. The colonies with more than 50 cells were counted. We divided colony number by plated cell number to calculate the colony forming efficiency (CFE, %). The experiments were repeated three times.

### Wound-healing assay

Approximately 2 × 10^6^ cells were seeded in 6-well plates. The cell monolayers were scratched using sterile 200-μl pipette tips after reaching 80% confluence. Serum-free medium was added into the plates after washing the floating cells. Cells were cultured at 37 °C for 48 h. The wound width was photographed and recorded every 24 h. The results were observed using the Image J software. Wound closure was computed according to the ratio of districts uncovered by cells before and after wound scratching.

### Cell invasion and migration assay

Transwell (8-μm pore size) chambers (Falcon) were coated with matrigel, placed in 24-well cell culture plates, and then air-dried in the incubator for 4 h. The 50 μL complete medium was added into each pore at 37 °C for 30 min. A2780/TCP1-NC and A2780/TCP1-KD cells were cultured with 5% FBS medium. Suspended cells (15 × 10^4^ cells/200 μL) were added into the upper chamber and 600 μl 15% FBS medium as a chemoattractant was put into the lower chamber. The invasive cells on the outside of the chamber were stained with 0.5% crystal violet after 48 h incubation. The cell slides were photographed under an inverted microscope in 5 randomly-selected fields at × 200 magnification.

For migration assays, the Transwell chambers were not coated with matrigel and the follow-up procedure was consistent with the invasion assays. Each experiment was conducted three times.

### Statistical analysis

All the data were represented as the mean ± standard deviation (SD). Statistical analyses were carried out by the SPSS software (version 20.0; SPSS) and Graph Pad Software (Graph Pad Prism 8.0.1). The independent *t*-test was used to the comparison of two groups. The one-way analysis of variance (ANOVA) followed by post-hoc test was applied for comparing multiple groups. *P* < 0.05 was considered as statistically significant.

## Results

### TCP1 was abundantly expressed in EOC tissues and its expression was significantly associated with the grade of differentiation and FIGO stage

**Fig. 1:**
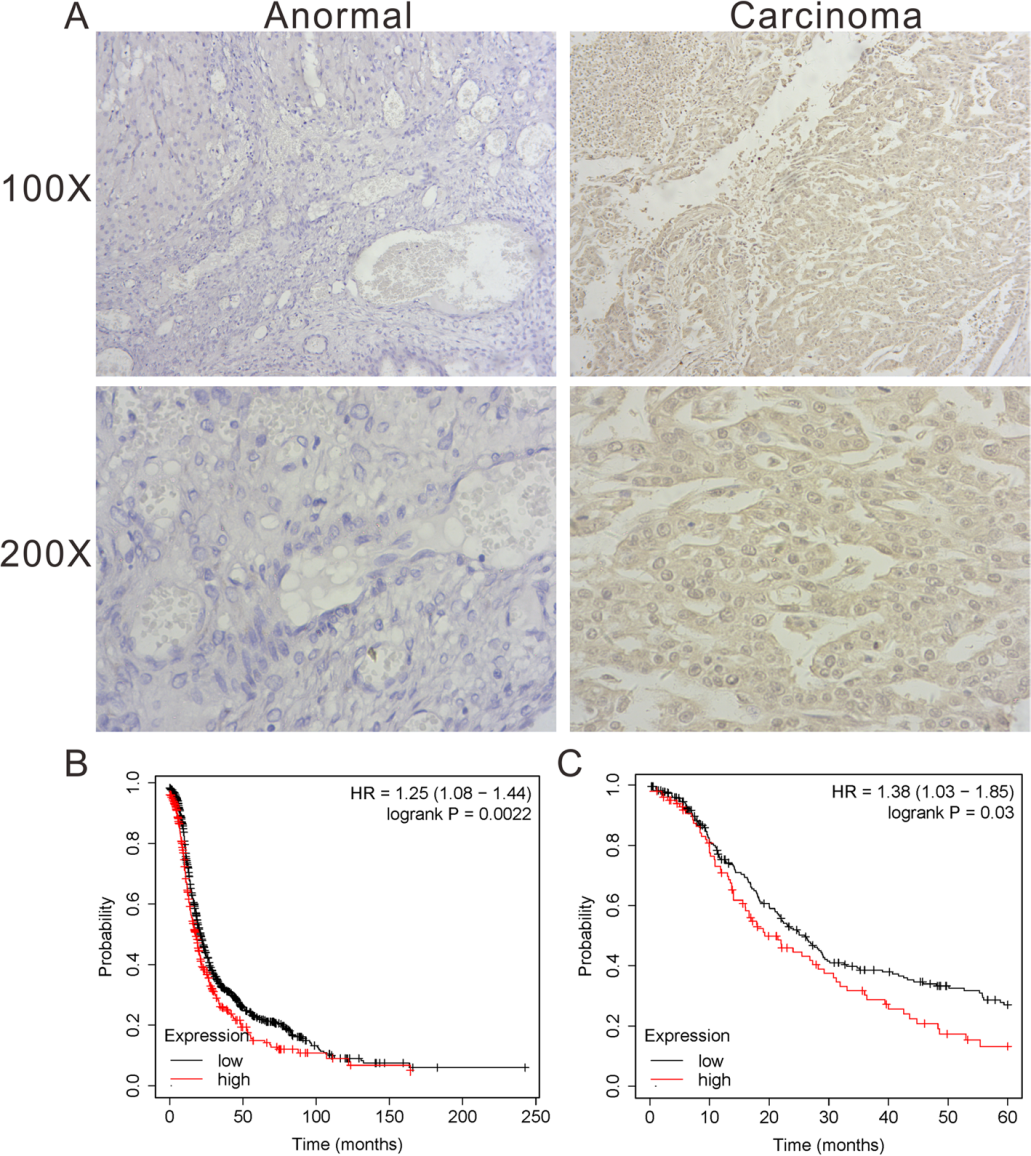
IHC analyses of TCP1 protein expression in different ovarian tissues specimens and Kaplan–Meier survival analyses of the EOC patients. **A** The expression of TCP1 in the normal and tumor samples detected by IHC. **B** The cut-off data was set as PFS, and the number of eligible cases in the database was 1435 KM Plotter. The HR value was 1.25 and the log-rank P was 0.0022. **C** The cut-off daata was set as OS, and the number of eligible patients in the database was 1656 KM plotter. The HR value was 1.27 and the log-rank P was 0.00088

Compared with non-EOC tissues, the staining intensity of TCP1 in EOC tissues was significantly higher, as per immunohistochemistry (IHC) assay (*P* < 0.05) (Table 2) (Fig. 1A). The relationships between the clinicopathologic variables of EOC and TCP1 expression are summarized in Table 3. ANOVA showed that the level of TCP1 expression correlated with the FIGO stage (the difference was statistically significant, *P* = 0.001) and the grade of differentiation (the difference was statistically significant, *P* = 0.001) but not with the tumor size, age, pathological type, lymph node metastasis, and volume of ascites. In summary, TCP1 upregulation may be associated with advanced stage of OC.

**Table 2:** The average optic density of TCP1 in ovary tissues in different histological subtype groups (IOD/area)

Group	n	IOD/area
Normal	13	0.003 ± 0.004
Cystadenoma	26	0.011 ± 0.015
Borderline	8	0.059 ± 0.050^a,b^
Carcinoma	62	0.077 ± 0.042^c,d^

Data presented as mean ± SD

^a^*P* = 0.001 vs. normal

^b^*p* = 0.001 vs. cystadenoma

^c^*P* = 0.000 vs. normal

^d^*p* = 0.000 vs. cystadenoma

**Table 3:** Relationship between TCP1 expression and clinicopathological features of EOC patients

**Characteristics**	**N**	**IOD/area**	***P***
Age (years)			0.271
< 60	47	0.074 ± 0.043	
≥ 60	15	0.087 ± 0.037	
Tumour size			0.72
< 5 cm	14	0.074 ± 0.037	
≥ 5 cm	48	0.078 ± 0.043	
FIGO Stage (2009)			0.001^*^
I + II	30	0.060 ± 0.033	
III + IV	32	0.094 ± 0.043	
Histological subtype			0.052
Serous	38	0.086 ± 0.044	
Mucinous	17	0.065 ± 0.026	
Clear cell	4	0.014 ± 0.007	
Endometrioid	3	0.091 ± 0.069	
Grade of differentiation^a^			0.001^*^
Low	27	0.050 ± 0.023	
High	35	0.091 ± 0.042	
Lymph node metastasis^b^			0.137
Positive	9	0.100 ± 0.048	
Negative	47	0.073 ± 0.040	
The volume of ascites			0.27
Positive	29	0.083 ± 0.039	
Negative	33	0.072 ± 0.044	

*N* Number of patients

^*^P < 0.05

^a^Low = G1; High = (G2 + G3)

^b^Additional six cases did not undergo retroperitoneal lymphadenectomy

### High expression of TCP1 mRNA led to a poor prognosis of patients with OC

We evaluated the prognostic significance of TCP1 in patients with OC using the Kaplan–Meier plotter analysis tool. We observed that patients with OC who had a higher TCP1 expression had a shorter progression-free survival (PFS) (HR = 1.25, *P* = 0.0022, *n* = 1453, Fig. 1B). High TCP1 mRNA levels were also associated with poor OS (HR = 1.27, *P* = 0.00088, *n* = 1656, Fig. 1C). The above results indicated that higher TCP1 mRNA levels predicted poor OS and PFS in patients with OC.

### Expression of TCP1 protein was upregulated in EOC cell line A2780

**Fig. 2:**
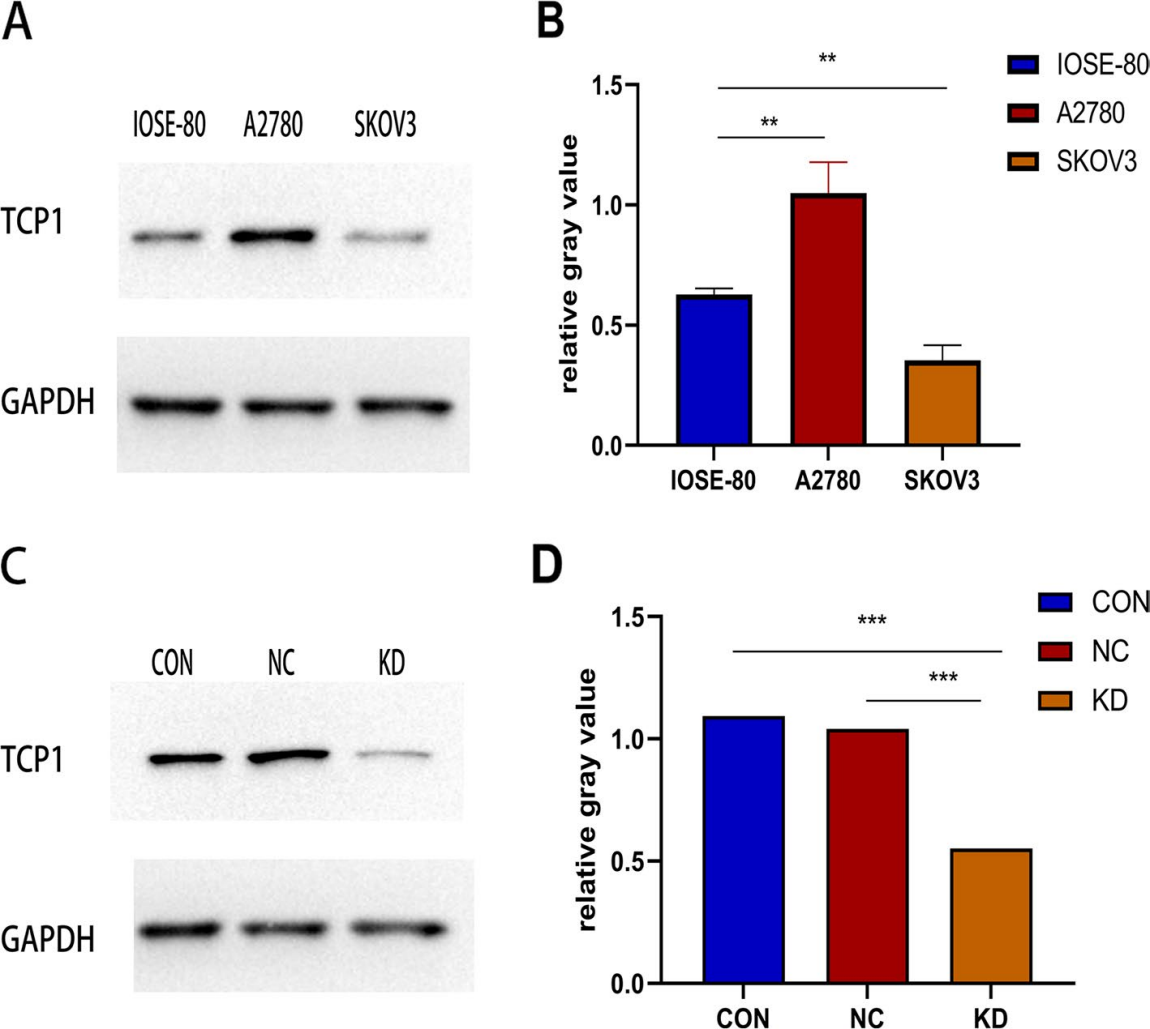
The expression level of TCP1 protein in human EOC cell lines as evaluated by western blotting and the knockdown effect by shRNA against TCP1. **A** TCP1 protein was up-regulated in A2780 cells, but not in SKOV3 cells, compared with IOSE-80 cells. **B** Histogram plotted with three relative gray values from **A**. Each cell line was conducted in triplicate. **P < 0.01 vs. IOSE-80. **C** The TCP1 expression levels of A2780/TCP1- KD cells that tested shRNA against TCP1 was markedly decreased, compared with A2780/TCP1-NC cells (NC: negative control) and A2780 cells control. (CON: controls). **D** Histogram plotted with three relative gray values from **C**. Each cell line was conducted in triplicate. ***P < 0.001 vs.A2780

TCP1 protein expression was evaluated by Western blotting in EOC cell lines A2780 and SKOV3. TCP1 was overexpressed in A2780 cells (*P* < 0.01) but not in SKOV3 cells, compared with normal cells IOSE-80 (Fig. 2A and B).

### TCP1 mRNA expression was inhibited in A2780 cells

Based on the different levels of two EOC cells detected by Western blotting (Fig. 2A and B), A2780 cells with high levels of TCP1 expression were selected for transfection with TCP1 shRNA lentivirus, and the stable TCP1 knockdown cells were cultured to study the function of TCP1 and its mechanism of action in EOC cells. After infection, knockdown of TCP1 protein expression in A2780 cells was confirmed by Western blotting (Fig. 2C and D).

### TCP1 knockdown inhibited cell growth in vitro

**Fig. 3:**
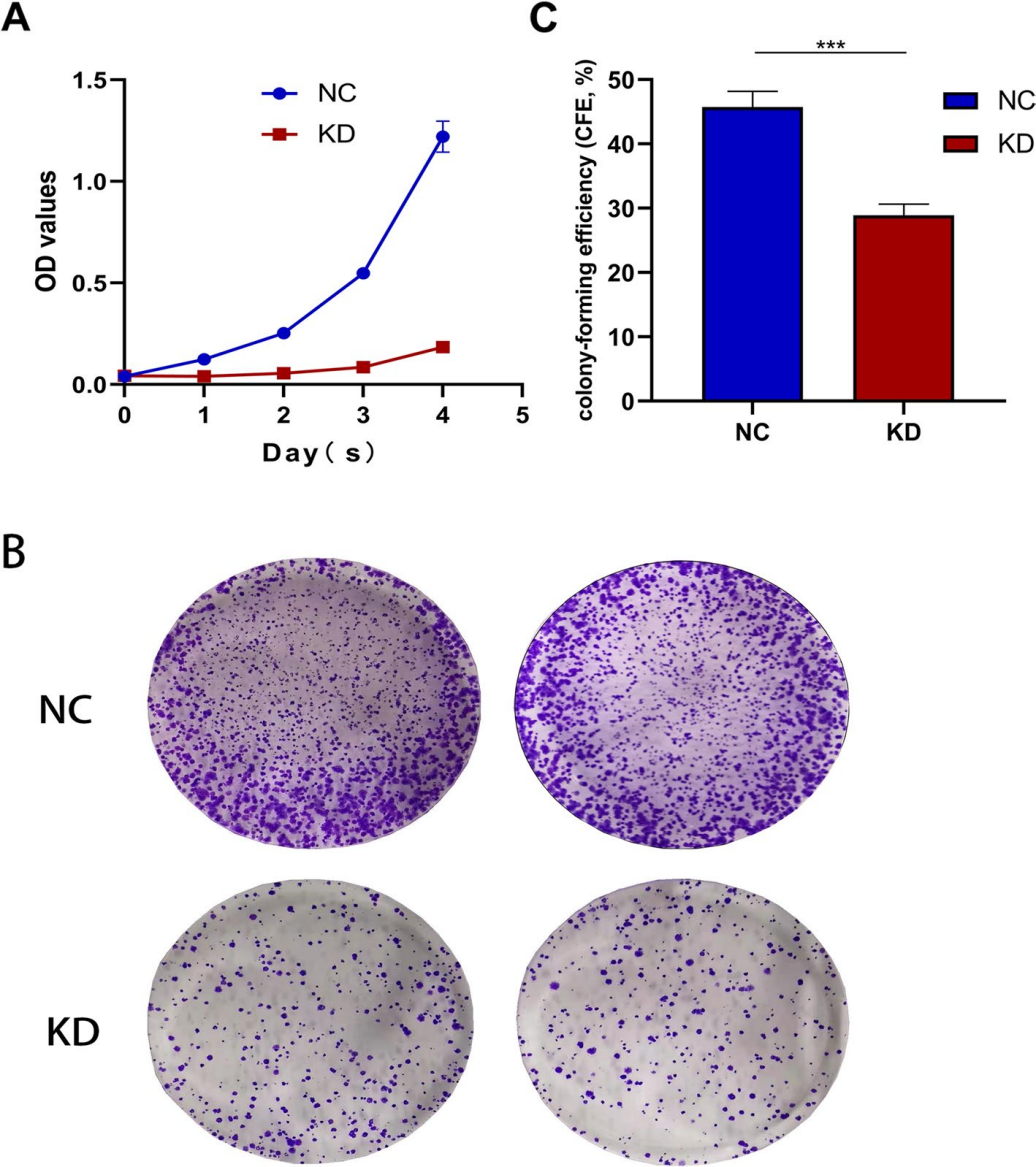
Growth-inhibiting role of knockdown TCP1 in A2780 cell lines. **A** The MTT assay showed that with a dramatic decrease of TCP1 expression, the proliferation of A2780 cells was significantly inhibited as shown by a nearly flat growth curve. The difference arose from Day 1 and persisted until Day 4. **B** Colony-formation assays indicated decreased growth rates in TCP1-KD A2780 cell line. **C** The colony-formation abilities from (**B**). One independent experiment was carried out in triplicate. Values are shown as the mean ± standard deviation (SD). Use independent Student’s t-test to calculate *P*-values. ***P < 0.001 vs. the NC

Cell proliferation assay using MTT and colony formation assays were performed to uncover the role of TCP1 in cancer cell growth. The cell viability of A2780/TCP1-KD was clearly suppressed compared with that of A2780/TCP1-NC (Fig. 3A); a similar finding was observed with regard to the colony-forming abilities (*P* < 0.01, Fig. 3B and C). The results of these assays demonstrated that TCP1 was essential for the growth of OC cells.

### TCP1 knockdown inhibited invasion and migration of A2780 cells

**Fig. 4:**
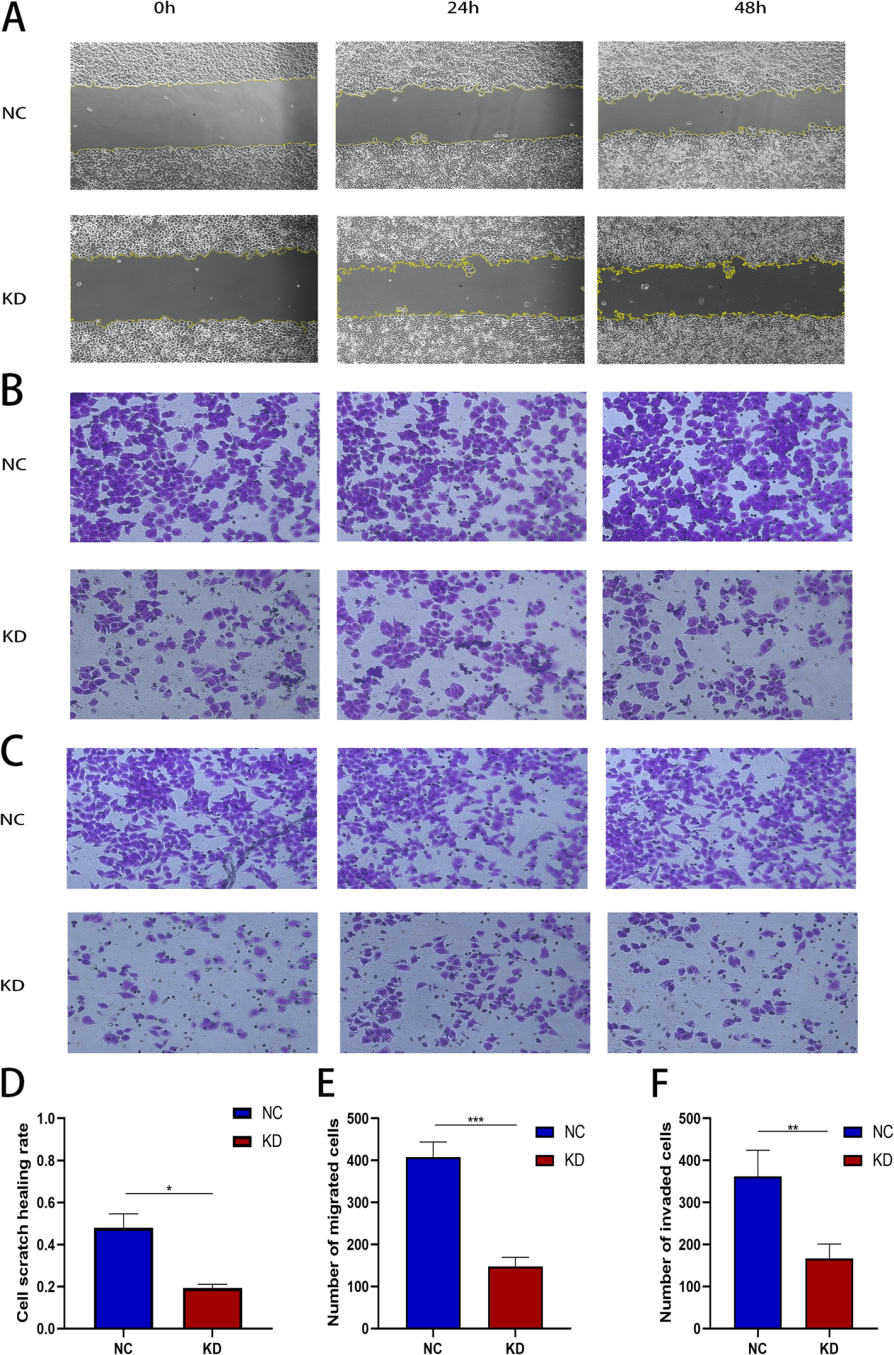
Suppression of EOC cell migration and invasion ability by TCP1 silencing. **A** and **D** TCP1 knockdown in A2780 cell line inhibited cell migration, as revealed by a wound healing assay. **B** and **E** Transwell assays revealed that shRNA-TCP1 knockdown decreased the migration of A2780 cells. **C** and **F** TCP1 knockdown remarkably attenuated the invasion ability of A2780 cells. Data are presented as the mean ± SD of three independent experiments. *P*-values were obtained with the independent Student’s t-test. *P < 0.05 vs. the NC; **P < 0.01 vs. the NC; ***P < 0.001 vs. the NC. Magnification: 200 ×

Wound-healing and transwell assays were performed to evaluate the migration and invasion abilities of A2780 cells after TCP1 silencing. The wound-healing assay showed that stable TCP1 knockdown inhibited the migration rate of A2780 cells, compared with controls (Fig. 4A and D). The transwell migration (Fig. 4B and E) and invasion (Fig. 4C and F) assays showed similar results. Collectively, these results demonstrated that TCP1 promoted cell migration and invasion in EOC.

### TCP1 regulated PI3K/AKT/mTOR signaling through p-AKT

**Fig. 5:**
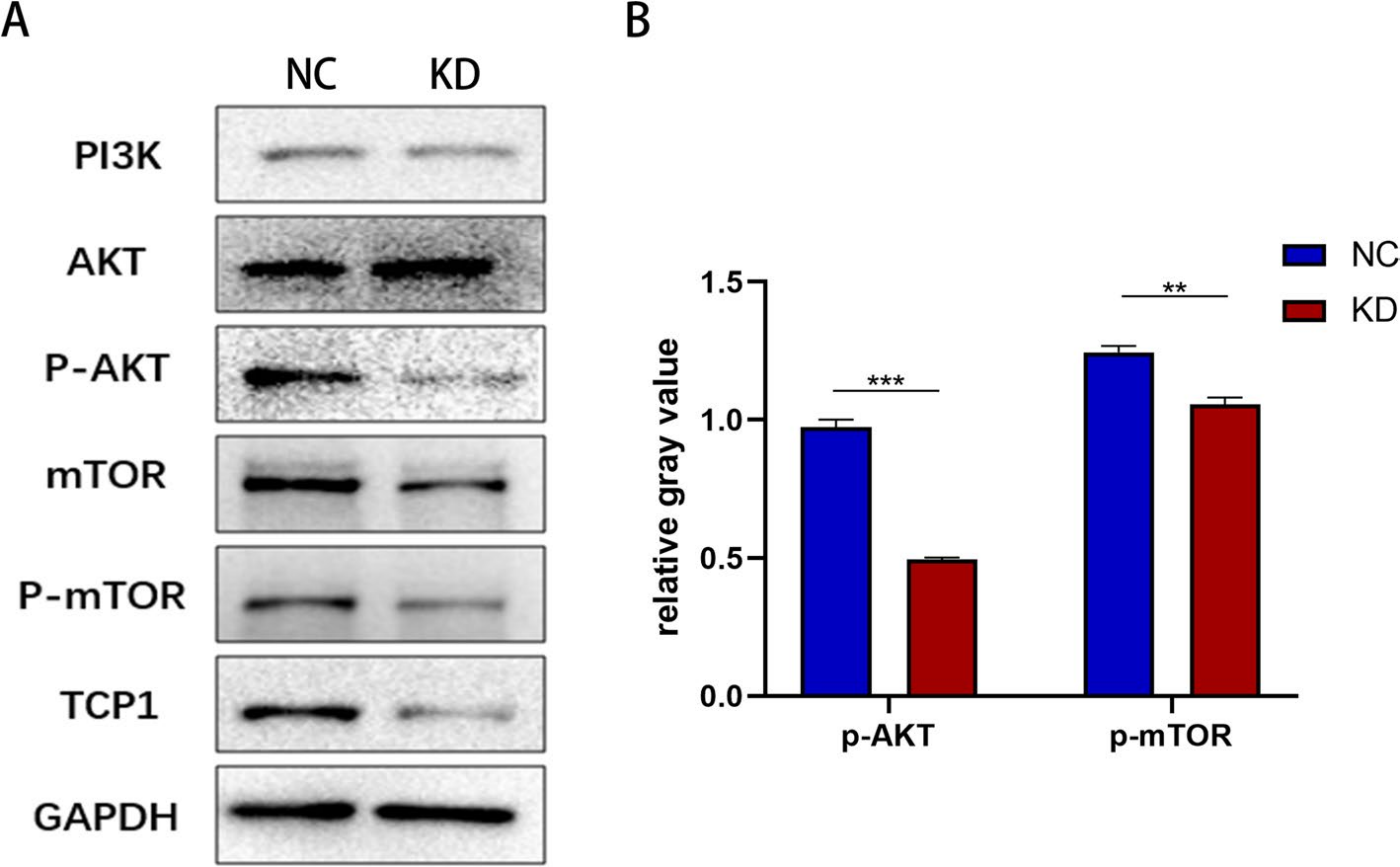
Knockdown of TCP1 inhibited PI3K/AKT/mTOR pathway. **A** Phosphorylation levels of PI3K, AKT and mTOR by Western blot analysis. The GAPDH was used as an internal control. **B** Histogram plotted with three relative gray values from (**A**). Data are presented as the mean ± SD of three independent experiments. **P < 0.01 vs. the NC; ***P < 0.001 vs. the NC

Protein was extracted from A2780/TCP1-NC and A2780/TCP1-KD cells. The results showed that phospho-AKT (p-AKT) and mTOR were decreased after TCP1 downregulation using Western blotting (Fig. 5A and B). GAPDH served as an internal control. Therefore, inhibition of TCP1 occurred via decrease of p-AKT in the PI3K/AKT/mTOR pathway to inhibit the development of OC, which was a central regulator of metabolism, survival, and proliferation in normal and cancer tissues.

## Discussion

Through this study, we demonstrated that TCP1 protein, a member of TRiC, is commonly altered in OC, essential for the development of OC cells, and related to the prognosis of patients with OC. Our study findings indicate the roles of TCP1 in OC and suggest that TRiC could be a novel target for the treatment of OC. The survival data of over 1000 patients collected from the KM plotter demonstrate that TCP1 upregulation is related to the poor prognosis of patients with OC compared with patients with wild-type expression of TCP1. To further determine the roles of TCP1 in clinical studies, the formalin-fixed paraffin-embedded samples were stained for TCP1 via IHC and TCP1 expression was analyzed. Interestingly, TCP1 was overexpressed in EOC samples, especially in higher tumor grade and higher FIGO stage samples. The findings suggested that patients with upregulated TCP1 expression had more aggressive OC. Inhibitors targeting TCP1 or TRiC complex may be beneficial for the prognosis of patients with OC with higher TCP1 expression, if TCP1 decisively affects their phenotypes.

TRiC, the most complex chaperonin, has eight distinct subunits encoded by different genes [12]. Originally, TRiC was identified and characterized by its essential role in folding cytoskeletal proteins such as tubulin and actin [14, 20, 21], cell cycle regulators Cdc20 [22], Cdh1 [22], p21ras oncoproteins [23], and the Von Hippel–Lindau tumor suppressor protein [3]. In addition, a previous study estimated that 5% of all cytoplasmic proteins in the eukaryotic cells are considered as components for the folding functions of TRiC [24]. Tubulins, one of the TRiC substrates, have been studied the most thoroughly and are the target of taxanes [25, 26], the common chemotherapy drugs for OC [27, 28]. Tumor resistance to taxanes is a challenge in the treatment of OC. The facts that TRiC is essential for folding of tubulin and patients with OC with upregulated TCP1 expression have poor survival suggest that TCP1 may affect the sensitivity of patients to taxanes. Future studies are warranted to clarify the underlying targets of TRiC to enhance the sensitivity of OC cells to taxanes, thereby overcoming resistance to the drugs.

Currently, there are no available data about the functions of TRiC or TCP1 in OC cells. Hence, we aimed to study the functions of TCP1 in the growth and survival of OC cells. We observed that TCP1 is differentially expressed in the various OC cells. For instance, TCP1 was upregulated in the A2780 cell line, but not in the SKOV3 cell line, compared with IOSE-80 cells, which is consistent with the findings of other studies [21]. This differential expression of TCP1 could be attributable to the fact that EOC has several pathologically distinct subtypes, such as endometrioid, serous, clear cell, etc. [29]; although the two cell lines used in this study were derived from patients with adenocarcinoma-type OC, they may have come from tissues with different subtypes while preserving their individual phenotypes. For instance, compared with the OC cell line A2780, the cell line SKOV3 showed tenfold greater resistance to cisplatin and 5.8-fold greater resistance to carboplatin [30]. In addition, TCP1 or TRiC activity is not always correlated with its expression level [21]. Therefore, we selected the A2780 cell line and successfully constructed stable cell lines of the A2780/TCP1-NC group and A2780/TCP1-KD group by using specific lentiviral shRNA targeting TCP1, which were used for further analysis. The functional studies confirmed that the silencing of TCP1 inhibited the motility and aggression of OC cells. The results suggested that TCP1 can accelerate the malignancy of OC, which is consistent with the findings of other studies that involved other tumors.

A previous study confirmed that the effect of TCP1 is achieved via the PI3K/AKT pathway [19]. Based on the recent studies on cancer genomics, several genes in the major cell signaling pathways were dysregulated in OC [8, 9]. Among these abnormal signaling pathways in OC, the PI3K/AKT/mTOR pathway was altered frequently, providing a great chance for developing a therapeutic intervention for OC [31, 32]. AKT, a known PI3-kinase target, is a serine–threonine kinase that regulates numerous downstream target genes [20, 33], ultimately regulating metabolic processes and cellular survival [16]. To investigate the underlying molecular mechanisms by which TCP1 improves the proliferation, invasion, and migration of OC cells, some proteins involved in the PI3K/AKT/mTOR signaling pathway were tested in A2780 cells. As shown in Fig. 5, compared with total AKT content, p-AKT level at serine 473 (Ser473), one of the two phosphorylation sites on AKT for its activation, was lowered in A2780/TCP1-KD cells [34]. Levels of p-AKT are typically determined to measure PI3K activity in cells. Altogether, the results showed that knockdown of TCP1 may inhibit the phosphorylation of AKT at Ser473 and its activation. In summary, TCP1 can regulate the proliferation, invasion, and migration of OC cells via the PI3K/AKT/mTOR pathway.

One possible limitation of this study is that the data were generated and analyzed from in vitro experiments. The specific mechanisms underlying the differential expression of TCP1 in the various OC cells and the possible regulation of p-AKT expression in OC by TCP1 warrant further investigation.

## Conclusion

In conclusion, TCP1 is overexpressed in OC and could be an important prognostic biomarker to predict the overall survival of patients with EOC. TCP1 may upregulate p-AKT, thereby promoting cell proliferation in OC.

## Supplementary Information

**Additional file 1.**

## Data Availability

The datasets of the overall survival (OS), progression-free survival (PFS), risk ratio (HR), and log-rank P of TCP1 in OC patients analyzed during the current study are available in the Kaplan–Meier Plotter repository, [http://kmplot.com]. The datasets of clinical parameter data used and analyzed during the current study are available from the corresponding author on reasonable request.

## Abbreviations

TRiC: TCP1 ring complex
TCP1: T-complex protein 1
OC: Ovarian cancer
EOC: Epithelial ovarian cancer
PI3K: Phosphatidylinositol-3 kinase
p-AKT: Phospho-AKT
OS: Overall survival
PFS: Progression-free survival
shRNA: Short hairpin RNA
shTCP1: Lentiviruses for TCP1 RNA interference
shCtr: Lentiviruses for negative control
