# Supplementary figures and images for: TCP1 regulates PI3K/AKT/mTOR signaling pathway to promote proliferation of ovarian cancer cells
Source: J Ovarian Res. 2021 Jun 23;14:82. doi: 10.1186/s13048-021-00832-x (PMC8223286; doi:10.1186/s13048-021-00832-x)

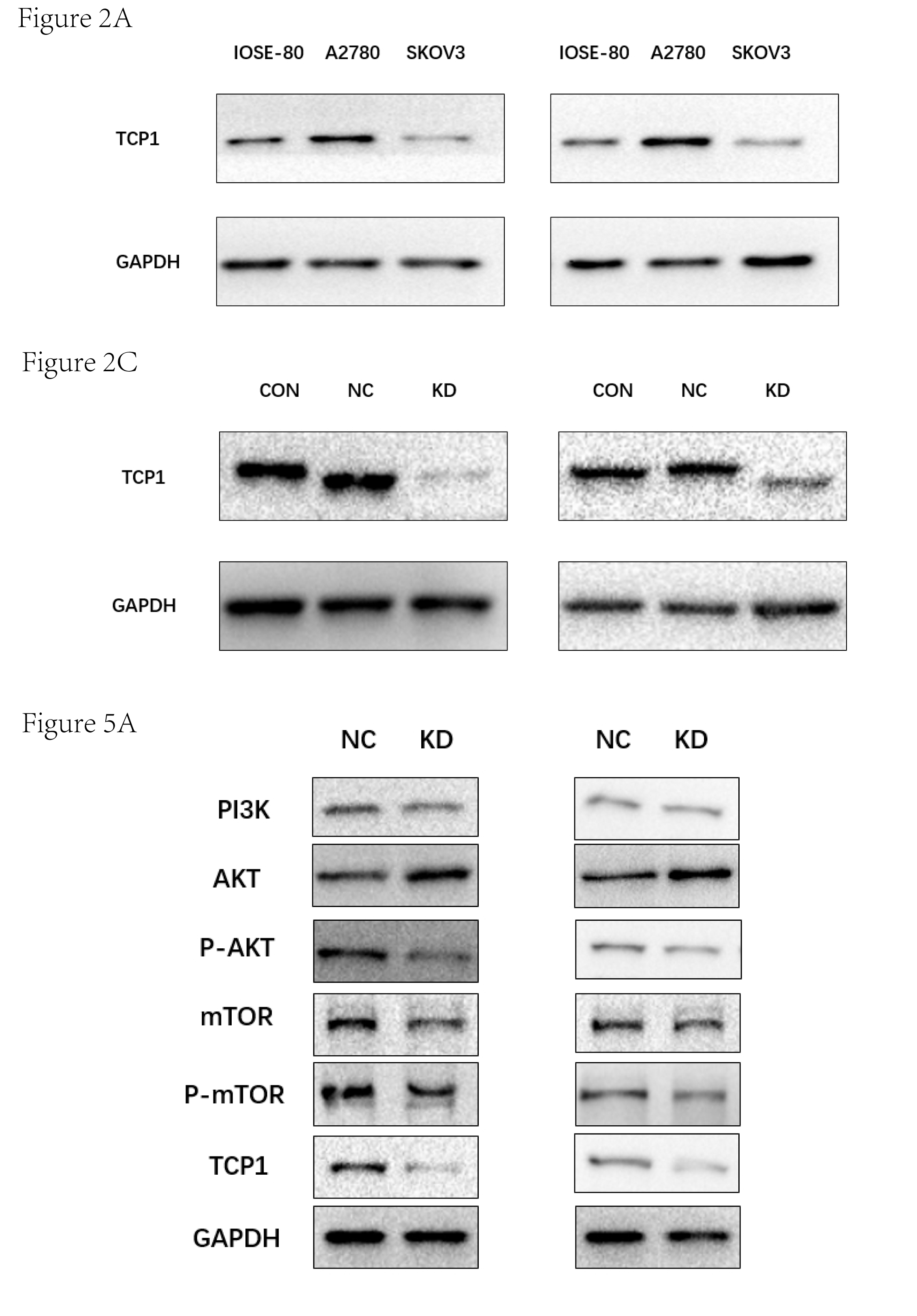

Supplement: Supplementary file 1 — Additional file 1. [file 13048_2021_832_MOESM1_ESM.tif]
